# Predictive Factors for Hospitalization of Patients with Heat Illness in Yamaguchi, Japan

**DOI:** 10.3390/ijerph120911770

**Published:** 2015-09-18

**Authors:** Takahiro Yamamoto, Masaki Todani, Yasutaka Oda, Tadashi Kaneko, Kotaro Kaneda, Motoki Fujita, Takashi Miyauchi, Ryosuke Tsuruta

**Affiliations:** 1 Advanced Medical Emergency and Critical Care Center, Yamaguchi University Hospital, 1-1-1 Minami-Kogushi, Ube, Yamaguchi 755-8505, Japan; E-Mails: tak-y@yamaguchi-u.ac.jp (T.Y.); todani-ygc@umin.ac.jp (M.T.); kaneyui-ygc@umin.ac.jp (T.K.); kouta-ygc@umin.ac.jp (K.K.); miyauchi-ygc@umin.ac.jp (T.M.); tsurutar@yamaguchi-u.ac.jp (R.T.); 2 Department of Acute and General Medicine, Yamaguchi University Graduate School of Medicine, 1-1-1 Minami-Kogushi, Ube, Yamaguchi 755-8505, Japan; E-Mail: motoki-ygc@umin.ac.jp

**Keywords:** heat illness, heat exhaustion, heatstroke, hospitalization, elderly, elevated body temperature, consciousness disturbance, dehydration

## Abstract

The objective of the study was to investigate the predictive factors for the hospitalization of patients who presented with mild to moderate heat illness at an emergency department. We conducted a retrospective survey of hospitals with an emergency department in Yamaguchi Prefecture, Japan. The survey questionnaire entries included patient age, sex, use of an ambulance, vital signs, blood examination conducted at the emergency department, the length of hospitalization, and outcome. We analyzed the predictive factors for hospitalization in patients with heat illness. A total of 127 patients were analyzed. Of these, 49 (37%) were admitted, with 59% discharged on the day following admission. In univariate analysis, the following inpatient characteristics were predictive for hospitalization: old age, low Glasgow Coma Scale score, elevated body temperature, increased serum C-reactive protein, and increased blood urea nitrogen. In logistic regression multivariate analysis, the following were predictive factors for hospitalization: age of ≥ 65 years (odds ratio (OR) 4.91; 95% confidence interval (CI) 1.42–17.00), body temperature (OR 1.97; 95% CI 1.14–3.41), Glasgow Coma Scale (OR 0.40; 95% CI 0.16–0.98), and creatinine (OR 2.92; 95% CI 1.23–6.94). The results suggest that the elderly with hyperthermia, disturbance of consciousness, and elevated serum creatinine have an increased risk for hospitalization with heat illness.

## 1. Introduction

With consideration of the phenomenon of global warming, an increased frequency of summer heat waves with extreme temperature elevation will have a strong influence on human health, heat illness in particular [1,2]. According to the report of the Intergovernmental Panel on Climate Change, the average summer temperature is currently on a rising trend internationally, and is increasing at a rate of approximately 0.6 °C per century [3]. Furthermore, the rate of global warming is increasing rapidly, with the maximum temperature during extremely hot summer days expected to increase by 2 to 3 °C in the next century [3]. High temperatures during the summer are associated with heat illness, including heatstroke; accordingly, the number of patients with heat illness visiting emergency departments has increased during hot summer days in recent years [4,5]. In most of these patients, the heat illness is self-limiting and they recover within several hours after lowering their body temperature, rehydrating, and resting, but some present with severe or prolonged symptoms such as deterioration of consciousness, fatigue, and headache [6,7]. In these patients, it may be necessary to treat organ failure caused by the heatstroke; however, the ultimate goal in the management of heat illness is to prevent exacerbation of the heat illness before organ damage can occur [8].

While heat illness occurs commonly in the summer, and is well known to the public, its definition remains somewhat confusing. Heat illness is the generic name for disease resulting from high environmental temperature or strenuous physical exercise [7]. In the medical context, the severity of heat illness is classified by the terms heatstroke and heat exhaustion. In the pathophysiology of heatstroke, inflammation and cell damage in patients exposed to heat stress have detrimental consequences that can lead to organ failure, such as rhabdomyolysis, renal failure, hepatitis, or coagulation disorder [9,10]. Because heatstroke complicated by organ failure has an unfavorable prognosis, it is essential to provide appropriate treatment without delay [11,12]. If heat exhaustion and heatstroke are considered to lie on the same spectrum, heat exhaustion can be regarded as a milder condition than heatstroke, and patients diagnosed with heat exhaustion can potentially develop heatstroke [11,13]. Therefore, it is extremely important to recognize those patients in the heat exhaustion phase whose illness is likely to progress to the more serious heatstroke.

The differential diagnosis of heatstroke and heat exhaustion can be very difficult because they are defined by body temperature ranges and nonspecific symptoms that overlap considerably, meaning that the pathophysiology and mortality are often not described accurately [11,13]. It is of concern that there are no solid criteria for determining the middle range of heat illness which would enable the differentiation between severe heat exhaustion and mild heatstroke. Accordingly, the decision to hospitalize the patient or not depends on the discretion of the physician. While much epidemiologic data exists relating to organ failure in patients with severe heatstroke, the risk factors for patients with potentially severe conditions, including heat exhaustion, have not been clarified. In this study, we report on a questionnaire survey conducted in hospitals with an emergency department in Yamaguchi Prefecture, with the aim of determining predictive factors for the hospitalization of patients with heat illness, focusing on those with heat exhaustion.

## 2. Materials and Methods 

### 2.1. Study Area 

This study was performed in 2007 in Yamaguchi Prefecture, Japan, which is located at latitude 34° N and has a population of 1.5 million people. The study data were collected from 1 July to 31 August, which is the period with the highest average temperatures of the year. We used the meteorological data measured by the Japan Meteorological Agency for the city of Yamaguchi, the centrally located prefectural capital in Yamaguchi Prefecture. The average temperature, average maximum temperature, and average minimum temperature over these two months for the past 30 years were 26.5 °C, 31.2 °C, and 22.9 °C, respectively [14].

### 2.2. Data Collection

The study was directed by the Advanced Medical Emergency and Critical Care Center at Yamaguchi University Hospital. We retrospectively conducted a research survey in each hospital in Yamaguchi Prefecture that has an emergency department. A survey questionnaire was sent via the Yamaguchi Medical Association to the 34 hospitals that participated in this study. Each patient diagnosed by a physician as having heat illness in these hospitals was registered in this study. The questionnaire was presented as a simple form, and was completed by each hospital with reference to the patients’ medical records. The items in the questionnaire were categorized according to patient characteristics, vital signs, laboratory data, and the presence or absence of hospitalization. The patient characteristics included age, sex, and whether the patient had traveled to the emergency department by ambulance. Vital signs included consciousness as measured by the Glasgow Coma Scale (GCS), blood pressure, pulse rate, body temperature, and respiratory rate. Laboratory data included the measurement of aspartate aminotransferase (AST), lactate dehydrogenase (LDH), C-reactive protein (CRP), creatine kinase (CK), blood urea nitrogen (BUN), creatinine levels, and complete blood cell count (CBC). The vital signs and the laboratory data were obtained at the time of the emergency department visit. The length of hospital stay and the outcome at discharge, including death, were also recorded in the questionnaire sheets. Death before admission was excluded from this study. These sheets were mailed or facsimiled to Yamaguchi University Hospital. The hospital name and identity of the individuals were not recorded in the database. Data was collected and analyzed by research doctors and the sheets were stored in a locked storage box. The Institutional Review Board of Yamaguchi University Hospital approved the study. The need for informed patient consent was waived because the non-interventional nature of the study posed no added risk to the subjects.

### 2.3. Statistical Analyses 

Comparisons of clinical features between hospitalized and non-hospitalized patients with heat illness were performed using the chi-square test and the Mann–Whitney U test. To identify the predictive factors for hospitalization in patients with heat illness, a multivariate analysis was performed by logistic regression with the stepwise method. A dummy variable for age was created that represented an age ≥ 65 and < 65 years, with the assumption of a situational difference between classic and exertional heatstroke. The effect of patient age on hospitalization was estimated by the dummy variable in the multivariate analysis. A *p*-value < 0.05 was considered statistically significant. Proportions are reported as an absolute number (%). Quantitative variables are reported as median (25th to 75th percentiles). Statistical analysis was performed using the statistical software Stat Flex version 6 (Artech CO., Ltd., Osaka, Japan).

## 3. Results

The mean maximum temperature in the city of Yamaguchi during the study period was 31.0 °C, and ranged from 23.4 °C (on 6 July) to 36.4 °C (on 17 August). The mean temperature and the mean minimum temperature were 26.6 °C and 23.5 °C, respectively. The total numbers of hot days and extremely hot days, as defined by the Japan Meteorological Agency as a maximum temperature exceeding 30.0 °C and 35.0 °C, respectively, were 40 days and eight days. There were two continuous periods of hot days in the study period, lasting 11 and 26 days. The mean temperature, the mean maximum temperature, and the mean minimum temperature during the study period were almost the same as the average temperatures recorded for the last 30 years [14].

**Figure 1:**
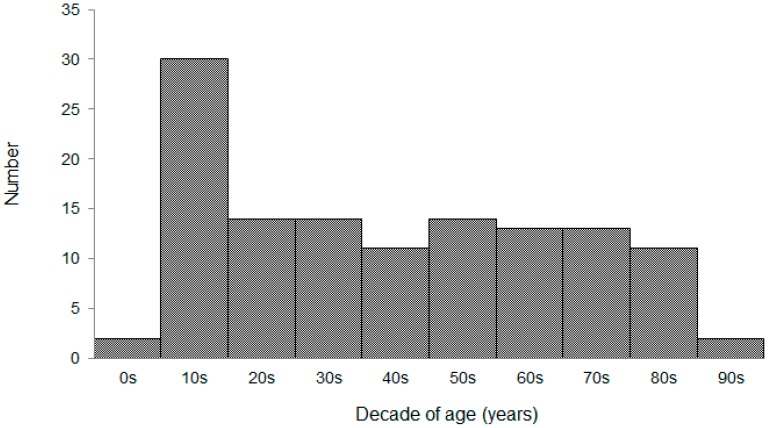
Ages of patients who presented with heat illness at the emergency department. Patient age ranged from five to 95 years, with teenagers presenting the most commonly.

Between 1 July and 31 August 2007, 128 patients presented with heat illness at the emergency departments of the study hospitals. One patient was excluded due to death during the emergency visit; therefore, a total of 127 patients were included in the analysis. The median patient age was 42 years and the inter-quartile range (IQR) was 19–63 years. Patient age ranged from five to 95 years, and more teenagers visited the emergency departments than any other age group (Figure 1). Of the 127 patients, 49 patients (39%) were admitted to the hospital. The median length of the hospital stay was two days (IQR, 2–5 days), with a maximum stay of 30 days. All of the hospitalized patients were treated successfully; 59% were discharged on the day following admission and 67% within three days of admission.

Table 1 shows a comparison of the clinical features between inpatients and outpatients with heat illness. The median age of inpatients was significantly higher than that of the outpatients (inpatient median 61 years (IQR 32–78), outpatient median 34 years (IQR 17–56), *p* < 0.01). The proportion of inpatients aged ≥65 years was significantly higher than the proportion of outpatients in that age group (inpatients, 47%; outpatients, 10%, *p* < 0.01). Although more males than females visited the hospital with heat illness, the gender difference between the inpatient *versus* outpatient groups was not significant (inpatients 71% male, outpatients 82% male, *p* = 0.16). There was also no significant difference in terms of hospitalization rates regarding whether the patients had traveled to the emergency department by ambulance (inpatient 45%, outpatient 37%, *p* = 0.39).

Among the vital signs, we did not analyze respiratory rates because of a lack of data (35% survey response rate). There was a significant difference in GCS between the inpatient and outpatient groups (inpatient median 15 (IQR 14–15), outpatient median 15 (IQR 15–15), *p* < 0.01). No significant differences in blood pressure or pulse rate were found between the two groups. Body temperature was significantly higher in the inpatients than in the outpatients (median 36.9 °C (IRQ 36.5–38.4) *vs.* 36.7 °C (IQR 36.3–37.3), *p* = 0.02), and only five inpatients had an elevated body temperature (>40 °C).

Biochemical blood analyses revealed no significant differences between inpatient and outpatient groups in terms of AST, LDH, CK, or creatinine levels. Significant differences between groups were found in terms of CRP (inpatient median 0.20 (IQR 0.10–0.46); outpatient median 0.06 (IQR 0.02–0.16), *p* < 0.001), and BUN (inpatient median 20.7 (IQR 14.3–24.6); outpatient median 15.4 (IQR 12.4–19.9), *p* < 0.01). In the CBC analyses, there were no significant differences between inpatients and outpatients in terms of white blood cell count, hemoglobin level, or platelet level.

A logistic regression analysis with stepwise methods was performed. The objective variable was hospitalization; the explanatory variables were age ≥65 years, sex, ambulance use, Glasgow Coma Scale, systolic blood pressure, pulse rate, body temperature, AST, LDH, CRP, CK, BUN, creatinine level, white blood cell count, hemoglobin level, and platelet count. Using a multivariate analysis, age ≥ 65 years (odds ratio (OR) 4.91; 95% confidence interval (CI) 1.42–17.00, *p* = 0.01), body temperature (OR 1.97; 95% CI 1.14–3.41, *p* = 0.02), Glasgow Coma Scale (OR 0.40; 95% CI 0.16–0.98, *p* < 0.05), and creatinine (OR 2.92; 95% CI 1.23–6.94, *p* = 0.02) were each found to be independent predictive factors for the increased likelihood of hospitalization (Table 2).

**Table 1:** Comparison of the clinical features between hospitalized and non-hospitalized patients with heat illness.

Clinical Feature	Inpatient (*n* = 49)		Outpatient (*n* = 78)		*p-*Value
Age (years)	61	(32–78)	34	(17–56)	<0.01
≥65 years	23	(47%)	8	(10%)	<0.01
Male	35	(71%)	64	(82%)	0.16
Ambulance use	22	(45%)	29	(37%)	0.39
Glasgow coma scale	15	(14–15)	15	(15–15)	<0.01
Systolic blood pressure (mm Hg)	127	(112–143)	120	(110–135)	0.25
Pulse rate (beats/min)	87	(78–102)	84	(71–91)	0.16
Body temperature (°C)	36.9	(36.5–38.5)	36.7	(36.3–37.3)	0.02
AST (IU/L)	30	(18–45)	24	(18–37)	0.33
LDH (IU/L)	270	(197–336)	232	(186–287)	0.06
CRP (mg/dL)	0.20	(0.10–0.46)	0.06	(0.02–0.16)	<0.01
CK (IU/L)	186	(132–522)	187	(126–381)	0.54
Creatinine (mg/dL)	1.04	(0.76–1.67)	0.90	(0.76–1.20)	0.35
BUN (mg/dL)	20.7	(14.3–24.6)	15.4	(12.4–19.9)	<0.01
White blood cells (/μL)	9300	(7400–12700)	8200	(6300–12000)	0.21
Hb (g/dL)	14.4	(12.7–17.3)	14.8	(13.4–15.7)	0.78
Plt (10^4^/μL)	23.6	(19.1–31.4)	23.4	(20.2–26.6)	0.56

Values are presented as the median and inter-quartile range, or absolute number and percentage.

**Table 2:** Independently associated factors for the hospitalization of patients with heat illness.

Factor	OR (95% CI) *		*p* Value ^†^
Age ≥ 65 years	4.91	(1.42–17.00)	0.01
Glasgow Coma Scale	0.40	(0.16–0.98)	<0.05
Body temperature	1.97	(1.14–3.41)	0.02
Creatinine	2.92	(1.23–6.94)	0.02
BUN	1.07	(0.99–1.15)	0.08

* OR: odds ratio, CI: confidence interval; ^†^ Multivariate logistic regression analysis (*n* = 127).

## 4. Discussion

The results of our study revealed several predictive factors for the hospitalization of patients who received medical treatment for mild to moderate heat illness at emergency departments. Age ≥ 65 years, elevated body temperature, deterioration of consciousness as measured by the GCS, and a high level of serum creatinine were all found to be predictive factors of patient hospitalization. These measurements can be used to detect a potentially severe condition in a patient during the early stage of heat illness, before the condition progresses. While much research has focused on severe heatstroke, few studies have examined the clinical features of mild heat illness. The results of this study document the clinical characteristics of patients with potentially severe heat illness and would help to identify patients with mild to moderate heat illness who should be observed carefully in emergency departments.

In our study, the age range was 5–95 years, with a predominant proportion of males. Teenagers presented much more frequently than patients in the other age brackets, with most other age ranges having similar numbers of patients (Figure 1). In general, heatstroke is divided into two categories, classic heatstroke and exertional heatstroke [10,13]. Classic heatstroke results from exposure to high environmental temperature and affects a socially vulnerable patient with advanced age and chronic medical conditions [15]. Previous epidemiological studies have shown that the elderly and those with chronic illness have an increased risk for hospitalization from heatstroke [11,16]. Exertional heatstroke is caused by strenuous exercise or activity, and affects young males most commonly [17]. Our data revealed that the majority of patients visiting an emergency department for heat illness were male teenagers, which suggests that most cases of heat illness could be classified as exertional heatstroke. With regard to the sex of the inpatients, there was a significant difference between the young and the elderly populations. Although most inpatients under 65 years old were male, in the group aged ≥ 65 years, the ratio of male-to-female inpatients was approximately equal (10 male and 13 female inpatients were aged ≥ 65 years; data not shown). These data suggest the importance of precaution in treating elderly people who meet the criteria for classic heatstroke.

The elderly are more susceptible to heat stress than the younger population due to their physical and social conditions [16,18]. Since the ability to acclimatize to thermal change declines with advancing age because of impaired physiological reserve capacity, the elderly are more affected by the rapid temperature changes that occur during heat waves [19]. One reason for the increased vulnerability of the elderly is their reduced heat dissipation capacity as a result of decreased skin blood flow, decreased sweat gland function, depletion of body fluids, and decreased cardiac output [20,21]. In addition, decreased thirst sensation and impaired renal function, which commonly occur in the elderly, are closely related to dehydration. In spite of the tendency of dehydration, inadequate fluid intake is often observed among the elderly [22,23]. These physiological factors observed as parts of the aging process can lead to dehydration, resulting in vulnerability under heat stress. While preexisting illness such as cardiovascular disease, diabetes, mental disorders, and insomnia are common in the elderly, not only the illness itself but also the medication used for treating the illness can increase the risk of severe heat illness [18,24,25]. This can be attributed to the physiological consequences of these medications, such as cardiac depressants and psychotropic drugs, which include an inability to increase cardiac output in response to heat, a decline in thermoregulatory ability, and decreased sweating [18,25]. Social factors in the elderly such as isolation from a social community, socioeconomic poverty, and decreased physical activity can also affect their vulnerability to heat illness because of delayed detection [1,16]. Japan faces the concern of a rapidly aging population, with the elderly aged ≥ 65 years currently accounting for approximately 25% of the total population of the country [26]. In a country in which society is aging, the elderly should be treated with consideration for their social and physical dispositions.

Hyperthermia and central nervous system dysfunction are major symptoms of heat illness [10,11]. The central nervous system is especially sensitive to heat stress [8,10]. Common symptoms in the early period of heat illness are anxiety, dizziness, fainting, and headache [7]. With progression to a pathological condition, the symptoms become more severe, with the patient exhibiting delirium, convulsion, and coma because the inflammatory response causes neuronal dysfunction, decreased cerebral blood flow, and increased intracranial pressure [8,9]. As the primary cause of organ injury, hyperthermia is the essential factor correlated with an adverse prognosis, resulting in multiple organ failures due to direct cell injury [27]. In our study, the body temperature of most patients was already under 40 °C, which is probably because the standard procedure performed by Japanese emergency medical services is to start cooling in the early phase when heat illness is suspected. Therefore, the body temperatures of our patients had probably already begun to decrease before they reached the hospital. In any case, the body temperature at the time of presentation to the emergency department is an important factor in making the decision for hospitalization, even if the patient has already started cooling before arriving at the hospital. Other information such as skin turgor, urinary volume, and the patient’s social background must be considered in the clinical setting [7,11,13]. To prevent the unfavorable consequences of a delay in starting treatment, measurement of body temperature and consciousness level are simple but important indicators of the severity of heat illness.

As organ failure can be a consequence of heat illness, it is especially important to detect organ failure, including rhabdomyolysis, renal dysfunction, liver injury, and an increased systemic inflammatory response, in the very earliest phases of heat illness [11,28]. Most biomarkers of organ failure are difficult to detect in the early phases of heat illness because of the time before the elevation of these biomarkers in the serum [7,29]. In our study, there were no significant differences between inpatients and outpatients in terms of the muscular and hepatic enzymes AST, LDH, and CK. It was difficult to use CRP as an index of inflammation for gauging heat illness severity, despite a significant difference between the inpatient and outpatient groups, because most CRP values fell below 1.00 mg/dL, an almost normal level, and CRP was not found to be an independent predictive factor. Renal function was the most sensitive and practical of the biomarkers for detection in the early phase of heat illness. Renal function is easily affected by the burden of heat stress caused by dehydration and rhabdomyolysis [13,30]. Furthermore, some investigators have suggested that patients with heat stress who have deterioration of renal function should be treated carefully because preexisting renal failure is one of the risk factors for a poor prognosis [31].

A limitation associated with our study is that we revealed only the predictive factors for hospitalization, meaning that the results do not accurately reflect the numbers of potentially severe patients because the relationship between the severity of heat illness and the length of hospital stay was unknown to this survey and all hospitalized patients survived. Although this study focused on patients with mild to moderate heat illness in the early phase, there is the possibility that immediate treatment of the patient in the emergency department prevented further exacerbation and unfavorable outcomes. It is well known that immediate treatment in the early phase of heat illness is the most effective method of improving the prognosis and avoiding residual sequelae [7,12]. While many studies have focused on cases of severe heatstroke with multiple organ failure, these have revealed little regarding the early phase of heat illness before the development of organ failure. The present findings are important because they document predictive risk factors of hospitalization that were determined in the early phase of heat illness in patients who visited emergency departments in various types of hospitals in a particular region.

## 5. Conclusions 

The results of the study showed that the predictive factors for hospitalization in patients with heat illness were age ≥ 65 years, hyperthermia, disturbance of consciousness, and elevated serum creatinine. These factors would help to identify patients with mild to moderate heat illness who should be observed carefully in emergency departments.
